# Cognitive behavioral therapy for a Japanese woman with olfactory reference disorder (ORD) comorbid with schizophrenia: A case study

**DOI:** 10.1002/pcn5.179

**Published:** 2024-03-08

**Authors:** Kazuki Matsumoto, Mari Nonaka, Kaoru Arai, Masayuki Nakamura

**Affiliations:** ^1^ Division of Clinical Psychology Kagoshima University Hospital Kagoshima Japan; ^2^ Department of Psychiatry Kagoshima University Graduate School of Medical and Dental Sciences Kagoshima Japan

**Keywords:** cognitive behavioral therapy, obsessive–compulsive disorder, olfactory reference disorder, olfactory reference syndrome

## Abstract

**Background:**

Olfactory reference disorder (ORD) is a mental illness in which individuals overestimate their sense of smell and worry about the negative impact of odors. Little is known about its successful treatment. A new cognitive behavioral model was developed based on cognitive behavioral therapy (CBT) for obsessive–compulsive disorder. Using this model, this study reports a successful treatment process of a 53‐year‐old female with ORD.

**Case Presentation:**

The patient's initial diagnosis was schizophrenia, and improvements were observed, such as the disappearance of persecutory delusions, through medication therapy. During this treatment process, it became clear that the patient's preoccupation with her own offensive body odor was not a hallucination or delusion caused by schizophrenia but rather a symptom of ORD. Within a limited 4‐week hospitalization period, high‐intensity CBT was provided by a clinical psychologist and a psychiatrist. Multiple CBT techniques were employed, including case formulation to identify her beliefs, reviewing safety‐seeking behaviors, attention shift training, behavioral experiments, public opinion polls, mindfulness meditation, and exposure and response prevention.

**Conclusion:**

Following a seven‐sessions intensive intervention over 3 weeks, her symptoms of ORD, anxiety, and depression reduced. High‐frequency CBT practices could be beneficial in treatment of patients with severe ORD, addressing severe ORD cases, facilitating rapid improvement in both ORD symptoms and functioning.

## Graphical abstract

While treatment for olfactory reference disorder (ORD) is not yet established, this case report suggests that cognitive behavioral therapy (CBT) is a promising therapeutic option. The CBT model for OCD is likely applicable to ORD. High‐frequency CBT practices could be beneficial in treatment of patients with severe ORD, addressing severe ORD cases, and facilitating rapid improvement in both ORD symptoms and functioning.

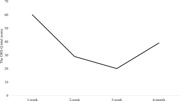

## BACKGROUND

Olfactory reference disorder (ORD) is characterized by an obsession with the belief that offensive odors of body or breath are grossly malodorous. Patients with ORD perceive themselves as highly malodorous, causing significant distress.^1^ They experience social impairment as they avoid interpersonal interactions,^2^ due to the fear of disturbing others with their odor.^3^ People with ORD engage in compulsive deodorizing behaviors,^4^ which are time‐consuming and disabling in daily life.^2^, ^5^ ORD typically has an adult onset and tends to persist if left untreated.^2^ Traditionally, the treatment for ORD has involved the use of selective serotonin reuptake inhibitors, clomipramine, or antipsychotic agents.^6^, ^7^, ^8^ The optimistic findings regarding these pharmacological treatments are based on a few case reports, and moreover, patients were not completely in remission. Another potential for ORD treatment is cognitive behavioral therapy (CBT). Some previous case reports have suggested that utilizing CBT adjusted for obsessive–compulsive disorder (OCD) models could potentially reduce ORD symptoms.^4^, ^9^, ^10^ The CBT model has been successful in treating female patients in the UK, the USA, and Australia.^4^, ^10^, ^11^ However, its applicability in East Asia, including Japan, has not been explored. Demonstrating effectiveness across cultural differences is crucial for a true feasibility evaluation of a specific psychotherapy.

In addition, the applicability of this CBT model for severe cases of ORD requiring hospitalization has not been tested. Intensive interventions are sometimes applied for patients with OCD, and this treatment approach is highly effective, much like typical frequency CBT in face‐to‐face settings.^12^ Particularly, for patients with severe ORD, high‐intensity CBT might be appropriate because in severe cases, it is often challenging to implement exposure and response prevention (ERP), which, independently, are central to CBT.^13^, ^14^ As mentioned above, there have been no reported cases applying intensive inpatient CBT for severe ORD. Exploring the potential of this approach holds significant clinical significance.

This study reports a successful treatment course of intensive CBT for a Japanese female patient with severe ORD during hospitalization. The intervention in this study primarily targeted cognitive processes, providing detailed information on specific beliefs, the approaches used for their modification, and compelling evidence for concentrated CBT in cases of a female Japanese patient with severe ORD symptoms.

## CASE PRESENTATION

“Naomi” (an alias) is a 53‐year‐old Japanese woman who was referred to the Division of Clinical Psychology, University Hospital, due to excessive and persistent concerns about smelling her hair, armpits, and feet. When she was a child, her family was poor and her clothes were dirty, so she was bullied for being “dirty” and having “odors.” From the time she was in middle school, she stopped attending school and became socially withdrawn. She had experience working at the company of her relatives and her father's acquaintances, but after a short period, interpersonal relationships did not go well, and she left the company. In her early thirties, surveillance delusions emerged, and she was diagnosed with schizophrenia. She was diagnosed with “drug‐induced scoliosis” when her spinal deformity developed during the period of taking olanzapine: she discontinued antipsychotics. For 13 years before the current admission time point, she entered a communal living facility and has been living there since while receiving welfare services. Both of her parents have died. She has friends in the facility and good relationships with the staff.

Naomi became obsessed with the idea that she was being slandered by the residents of the facility about her own “offensive odors” and increased her camouflage behavior using Duo Durand, antibacterial sheets, and deodorant spray, among other methods. She began to wash her body in the bath more frequently and for longer duration and tried to further increase the number of baths taken; however, the rules of the facility prevented her from doing so because of the COVID‐19 pandemic. Naomi became overly concerned about her own stench, and she quickly became distraught, often crying, “I can't do anything.” The facility staff and her sister encouraged Naomi to admit herself to the hospital. After agreeing to intensive care, Naomi was admitted to a regional general hospital. During her hospitalization, she bathed every other day, approximately three times a week. This bathing frequency was consistent with the facility she was originally in during the COVID‐19 pandemic.

In the first week of her hospitalization, assessments were conducted to measure the severity of various factors, including brain imaging, cognitive function, and psychotic disorders. Magnetic resonance imaging showed no abnormalities, and the Wechsler Adult Intelligence Scale IV indicated an average intelligence quotient (full scale IQ) of 93. The attending psychiatrist (MNo) diagnosed schizophrenia and initiated treatment with brexpiprazole 0.5 mg during the first week of admission on June 13th, which was increased to 1.0 mg the following week on June 20th. The pharmacotherapy significantly alleviated Naomi's persecutory delusions, characterized by the belief that her belongings were being stolen and she was being mistreated. In contrast, her strong conviction that she had a “bad smell” remained unchanged, leading to continued behaviors, such as camouflage, avoidance of social situations, frequent changing and cleaning, and self‐checking her offensive odor. This condition met the criteria for ORD as per the *DSM‐5‐TR* diagnosis.^15^ The attending psychiatrist (M. No.) asked a clinical psychologist (K. M.) specializing in CBT to provide psychotherapy for ORD.

An intervention consisting of seven intensive sessions of individualized CBT was arranged during a 4‐week hospital stay. All sessions were set for 60 min to implement cognitive–behavioral techniques that addressed Naomi's symptoms. CBT modules included case formulation to identify her beliefs, reviewing safety‐seeking behaviors, attention shift training, mindfulness, relaxation, behavioral experiments, public opinion polls, and ERP. All sessions were conducted by a trained CBT therapist/clinical psychologist (K. M.). The attending psychiatrist (M. No.) managed the pharmacotherapy and supported exposure and response prevention methods in the ward. A supervising psychiatrist (K. A.) provided guidance to the attending physician's treatment approach and managed the case, while a senior psychiatrist (M. Na., the senior author) organized the psychiatric care team and coordinated overall medicine administration for Naomi. Please see the supplementary file for more detail on the practice of CBT.

**Figure 1:**
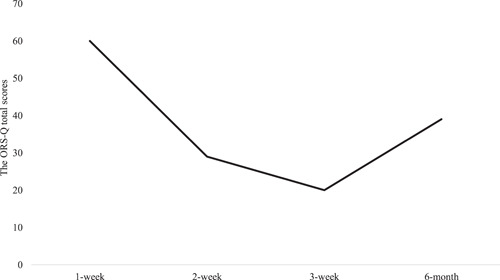
The Olfactory Reference Syndrome Questionnaire (ORS‐Q) total scores at the 1‐week, 2‐week, 3‐week, and 6‐month follow‐up.

The outcomes for symptom assessment utilized the Yale–Brown Obsessive–Compulsive Scale (Y‐BOCS),^16^, ^17^ the Olfactory Reference Syndrome Questionnaire (ORS‐Q),^4^ the Patient Health Questionnaire 9‐items (PHQ‐9),^18^, ^19^ and the Generalized Anxiety Disorder 7‐items (GAD‐7).^20^ Please refer to the supplementary files for descriptions of each scale. Table 1 shows the outcomes at the baseline (first week of hospitalization), second week, third week (at the conclusion of CBT), and 6‐month follow‐up. At the time of discharge, ORD symptoms had significantly decreased (Figure 1). Naomi's bathing duration reduced from over an hour to less than 30 min, the number of times she washed her body decreased from three times to once per day, and her changing of clothes reduced from four times to once per day. She no longer avoided social interactions and could often engage in conversations with friends made within the ward. However, the belief that she emitted a foul smell persisted. Interestingly, a significant change was observed in her ability to doubt whether her conviction of smelling bad was accurate. Naomi expressed, “I believe I smell, but people around me say ‘you don't smell that bad,’ so maybe it's not as severe as I think.”

**Table 1:** Outcomes at 1‐week, 2‐week, 3‐week, and 6‐month follow‐up.

Variable	1‐week	2‐week	3‐week	6‐month
Y‐BOCS	34	23	11	15
ORS‐Q	60	28	20	39
PHQ‐9	18	11	1	11
GAD‐7	11	7	1	6

Abbreviations: GAD‐7, Generalized Anxiety Disorder 7 items; ORS‐Q, Olfactory Reference Syndrome Questionnaire; PHQ‐9, Patient Health Questionnaire 9 items; Y‐BOCS, Yale–Brown Obsessive–Compulsive Scale.

At the 6‐month follow‐up, we observed an increase in Naomi's scores for the Y‐BOCS, ORS‐Q, PHQ‐9, and GAD‐7. During this period, a case of COVID‐19 was confirmed within the communal living facility where she resides. This event likely introduced significant stress and anxiety, potentially causing a transient exacerbation of her obsessive–compulsive symptoms.

## DISCUSSION

Throughout the treatment process, the cognitive–behavioral model developed by Allen‐Crooks and Challacombe (2017) was applied intensively upon admission.^4^ The pharmacotherapy, initiated shortly after her hospitalization, provided emotional stability, enabling her to engage in CBT twice a week. Within CBT, specific techniques, such as the reaction blockage method, which curtailed excessive cleaning, behavioral testing to assess perceived odor threat levels, discontinuation of camouflage behaviors (safety‐seeking behavior), and addressing reasoning biases, were performed and alleviated Naomi's ORD symptoms. The technique derived from Clark and Wells's (1995) model,^21^ including polling methods, played a pivotal role in modifying Naomi's ORD beliefs.

This case report represents the world's inaugural effort to administer CBT for ORD more frequently than twice a week. Time‐intensive treatments are also considered in cases of refractory OCD, offering a higher level of care within a shorter timeframe, contrasting with traditional weekly hour‐long sessions.^12^ Intensive CBT is a therapeutic approach that involves providing CBT for extended periods, typically ranging from 1 h to a maximum of 8 h. The time constraints that prevent the delivery of such extended interventions to a single patient are common in general hospitals or psychiatric facilities that are not research oriented. However, there are instances where high‐frequency interventions are feasible, and this case is one such example. The effectiveness of providing interventions at high frequencies is largely unknown and warrants further investigation. Intensive CBT also showed superior short‐term improvement in the treatment of OCD; however, long‐term follow‐ups have indicated reduced treatment efficacy.^22^, ^23^ The findings align with Naomi's treatment course, suggesting that intensive CBT for ORD may follow a similar trajectory as in OCD. A previous study tracking symptoms of OCD patients 8–10 years after inpatient ERP‐based treatment reports that while obsessive–compulsive symptoms continue to improve, the remission rate is 20%, indicating a low level of improvement compared with immediately after treatment.^24^ The study concludes that sustained improvement relies on the ongoing practice of exposure training. Maintenance of exposure training could help patients with ORD achieve a favorable prognosis for the long term.^4^

Delusions observed within obsessive thoughts are often misconstrued as originating from schizophrenia.^25^, ^26^ According to Hudak and Rasmussen,^27^ there is no evidence supporting insight, oddness, and overvalued ideas as distinguishing factors between delusion in schizophrenia and obsessive thoughts in OCD. They argue that patients' reactions to contradictory evidence are more helpful in differentiation.^27^ In schizophrenia, where prominent delusions are observed, patients typically incorporate contradictory evidence into their delusional systems. In the case of OCD, patients demand more evidence continually, as it serves the pursuit of reassurance. From this perspective, given Naomi's persistent search for evidence that her smell is harmless, her condition can be considered obsessive thoughts. We believe our diagnosis of Naomi's complex and challenging clinical syndrome as paranoid delusions in schizophrenia and ORD, respectively, is accurate. This case teaches us the importance of carefully diagnosing complex cases.

Schizophrenia was managed by the attending physician, and Naomi had good adherence to medication. The prevalence of obsessive–compulsive and related disorder in schizophrenia is 10%–24%.^28^, ^29^ Patients with those morbidities often exhibit higher levels of anxiety, depression, and psychotic symptoms.^30^ In the *DSM‐5‐TR*, ORD is classified under obsessive–compulsive and related disorders.^15^ In this case, both schizophrenia and ORD coexisted, significantly impairing the patient's daily life and social functioning. Evidence from a study conducted on Chinese female schizophrenia patients showed a relationship between positive symptoms and olfactory identification.^31^ In the present case, pharmacotherapy targeting positive symptoms of schizophrenia was implemented, potentially enhancing olfactory identification, and indirectly promoting CBT for ORD. In fact, Naomi stated that pharmacotherapy made it easier for her to engage in CBT. This case report suggests that pharmacotherapy preceding CBT and intensive psychosocial interventions may contribute to treating patients with both schizophrenia and ORD, suggesting the potential benefits of such an approach.

## CONCLUSION

This study provides evidence that time‐intensive CBT significantly reduces the core symptoms of ORD in the short term. Future studies should concentrate on evaluating the efficacy of time‐intensive CBT, incorporating validation in larger samples through randomized controlled designs. This CBT protocol for ORD holds promise for adaptation both in Western and Eastern cultures.

## AUTHOR CONTRIBUTIONS

Kazuki Matsumoto designed the study, conducted the interventions, and drafted the manuscript. Mari Nonaka designed the treatment during hospitalization and implemented pharmacotherapy. Kaoru Arai provided clinical supervision for the case. Masayuki Nakamura and Kaoru Arai contributed to and approved the final manuscripts.

## CONFLICT OF INTEREST STATEMENT

The authors declare no conflicts of interest.

## ETHICS APPROVAL STATEMENT

The authors have abided by the Ethical Principles of Psychologists and Code of Conduct as set out by the BABCP and BPS.

## PATIENT CONSENT STATEMENT

We obtained informed consent from the individual through a research explanation provided verbally and in writing.

## CLINICAL TRIAL REGISTRATION

None.

## Supporting information

Supporting information.

Supporting information.

Supporting information.

## Data Availability

The authors confirm that the data supporting the findings of this study are available within the article.
