# Supplementary material for: Cognitive behavioral therapy for a Japanese woman with olfactory reference disorder (ORD) comorbid with schizophrenia: A case study
Source: PCN Rep. 2024 Mar 8;3(1):e179. doi: 10.1002/pcn5.179 (PMC11114287; doi:10.1002/pcn5.179)
Supplement: Supplementary file 1 — Supporting information. [file PCN5-3-e179-s003.docx]

**Supplemental file**

**Cognitive behavioral therapy for a Japanese woman with olfactory reference disorder (ORD) comorbid with schizophrenia: a case study**

**Case-formulation based on Salkovskis’ (1985) model**

We employed a framework developed for ORD based on Salkovskis' 'vicious flower formulation' (1985).^1^ The CBT model was organized and conceptualized around a recent incident in which Naomi was concerned about her own odor (Fig. S1). In this case, we observed that in Naomi's hospital room, there was an intrusive thought, "My hair and body bad smells," and that the central meaning focused on the avoidance of others and safety-seeking behavior, including camouflage. In addition, we also noticed that the memory of being bullied for offensive odors in childhood formed the assumption that a person with offensive odor is hated. Naomi used the facial expressions and behaviors of others in social situations as cues for interpreting that she was dirty and smelly. She frequently asked others about her odors. To deal with her odor, she used deodorant sprays and Duo Durand, wiped the sweat, washed her body to 3–4 times while bathing, and changed her clothes four times a day. It was identified that underlying these safety-seeking behaviors was a catastrophic belief that if she does not deal with odors, she will be harassed or ostracized by others.

**Fig S1. Here.**

**Couse of cognitive behavioral therapy**

The focus of session 1 was on assessment interviews and case conceptualization on June 22. Symptom severity was assessed using the aforementioned outcomes and Naomi was provided with immediate feedback on the results. After a case map was drawn, techniques were introduced to help her improve her symptoms, and response obstruction was introduced step-by-step; unexpectedly, the feared object she repeatedly avoided could not be located. The lack of hand sanitizers, antiperspirants, and deodorants was the first reaction-blocking challenge. The need to stop obsessional rumination was also addressed. Instead of obsessional rumination, she chose an alternative behavior of 'watching live footage of Korean idol singers.’

In session 2 on June 24, the cognitive bias “identification of thinking and reality” was introduced, and we examined whether it applies to Naomi. Alternative explanations for Naomi's self-odor-related problems were explored using theories A and B. As a result of this study, the following theories are proposed:

Theory A. People hate me and are ostracized because of my offensive odor.

Theory B. I am very worried that people will hate me because of my smell.

To examine the validity of these theories experimentally, the first behavioral experiment was designed. We decided to observe whether the reaction from surrounding people would change negatively by changing clothes only after bathing instead of changing clothes to 3–4 times a day. In the behavioral experiment, no negative behavior was observed in the reactions of patients in the same room as hospital staff.

Session 3 dealt with excessive worry about slandering on June 27. Through exposure to worry, the cognitive-behavioral technique is frequently used in the treatment of generalized anxiety disorder (McIntosh & Crino, 2013), spending time in the ward in a room where patients can interact with other patients and accept intrusive thoughts. Then, patients practice mindfulness meditation and acceptance. Relaxation techniques, such as breathing exercises and progressive muscle relaxation, have been introduced to help maintain exposure without avoiding anxiety/fear. After the session, homework was assigned to practice the techniques instead of reacting to intrusive thoughts in the ward.

In Session 4 on June 30, Naomi reported increasing activities that induce positive feelings, such as reducing worries and engaging in video watching. An attention-shifting technique was introduced when excessive attention was directed towards one's own smell or worries. This technique aimed to enhance the flexibility and control of attention by suppressing excessive immersion in strong worries (Barth *et al.* 2019). Naomi practiced mindfulness meditation with her CBT therapist (KM), letting intrusive thoughts pass by, both in her hospital room and in the counseling room.

In Session 5 on July 6, we took advantage of hospitalization in the middle of summer to challenge her fear of sweating. In the area where our treatment facility is located, the temperature in summer remains over 30 °C during the day. In this environment, we strolled around the hospital grounds, naturally triggering sweating. Naomi conducted an opinion poll for her offensive odor with three nurses (two females and one male), a resident doctor (female), and a clinical psychologist (male). In the polls, no one grimaced at Naomi; instead, they approached her face. After this work, her alertness to sweating significantly reduced. A poll was conducted to find out what percentage of people followed Naomi's belief that “people with odors be hated, I'm dead sure.” Polling, a technique used in CBT for social anxiety disorder (Clark & Wells, 1997), is a systematic survey of how individuals with social limitations interpret their surroundings and others. We asked the opinion of the five aforementioned medical staff members, whose average age was late twenties, and no one agreed with the beliefs. Their response influenced lowering the threat level of smell, and Naomi began to think that “people with odors not necessarily are hated." These cognitive-behavioral techniques have reduced the probability of Naomi's belief "I am emitting a bad smell" from 100% to 50%.

In session 6 on July 11, we dealt with Naomi's inference bias that 'everyone hates me of offensive odors' and attention bias toward other people's negative behavior. Her childhood was often discriminated against because she was not very athletic, and her hands were clumsy. Her parents were very poor so she was unable to bathe in the summer, and she explained her experience of having her classmates point out her foul odor. These experiences likely led her to associate someone's stench with her and interpret it as disgust when someone twisted their face or clicked their tongue. Through a cognitive restructuring procedure, Naomi noticed that other people's behaviors, which indicate their concerns about odors, may have been caused by other factors.

***Therapist:*** “When you smell an offensive odor, do you automatically distort your face? No other reasons?”

***Naomi:*** “Even if it does not smell bad, there are times when my face is distorted. For example, when something bad occurs, you feel sad.”

***Therapist:*** “Yes. Perhaps it is not about Naomi's smell but about job. The dog may have recently been sick.”

***Naomi:*** “OK, I can think of it that way. I do not know if I can do it, but I will try.”

The homework for Session 5 was to think about other factors in the process of cognitive restructuring instead of attributing the cause to one's own stench when noticing someone else's negative behavior. This includes the discontinuation of excessive attention.

In Session 7 on July 13, Naomi worked on cognitive restructuring to coping with her negative thoughts that were maintaining her depressive mood. Naomi had decided to move to a new facility after discharge, and she was concerned about living in that new environment. She introduced the cognitive restructuring techniques frequently used in cognitive therapy for depression (see Table S1 for details). Through role-playing, Naomi experienced that worrying excessively actually increased her anxiety rather than providing comfort.

**Table S1. Here.**

**Outcomes in the current case**

The primary outcome of this study was the Y-BOCS total score, which measures OCD severity.^2,3^ The Y-BOCS consists of 10 items, each of which scored on a Likert scale from 0 to 4 (range 0–40). The severity of ORD was assessed using the Olfactory Reference Syndrome questionnaire (ORS-Q), as utilized in previous research.^4^ The ORS-Q is a 24-item scale that measures ORD-related distress, beliefs, and behavioral problems. The measure requires respondents to rate how frequently the item has occurred over the past week on a five-point Likert scale: 'not at all,' ‘a little,’ 'often,’ ‘a lot, or ‘all the time.’ The validity and reliability of the ORS-Q have not yet been confirmed; it was translated into Japanese by the first author and used in this study.^3^

To measure depressive and general anxiety symptoms as comorbidities, Japanese version of the Patient Health Questionnaire 9-items (PHQ-9) and the Generalized Anxiety Disorder 7-imet (GAD-7) were used, respectively.^5-7^ The PHQ-9 is a scale developed to assess the severity of depressive symptoms and consists of nine questions with answers ranging from 0 'not at all' to 3 'nearly every day'. In terms of the clinical meaning of the PHQ-9 total score, 0–4 is perceived as ‘none-minimal,’ 5–9 is 'mild,’ 10–14 is 'moderate,’ 15–19 is 'moderately severe,’ and 20–27 is 'severe.’ The GAD-7 is a scale developed to assess the severity of anxiety symptoms and consists of seven questions ranging from 0 (‘not at all’) to 3 (‘nearly every day’). In terms of the clinical meaning of the GAD-7 total score, 0–4 is considered ‘minimal anxiety,’ 5–9 is 'mild,’ 10–14 is 'moderately,’ and a score >15 is 'moderately to severe,’ and 20 or more is 'severe.’ The PHQ-9 and GAD-7 are well-validated measures that have been identified as clinically important across depression and anxiety disorders, respectively. Their validity and reliability have been confirmed in Japan.^4^

**References**

1. Salkovskis PM. Cognitive-behavioural problems: A cognitive-behavioural analysis. Behaviour Research and Therapy. 1985; 23:571-583.
2. Goodman WK, Price LH, Rasmussen SA, et al. The Yale-Brown Obsessive Compulsive Scale. I. Development, use, and reliability. *Arch Gen Psychiatry*. 1989;46(11):1006-1011. doi:10.1001/archpsyc.1989.01810110048007
3. Nakajima T, Nakamura M, Taga C, et al. Reliability and validity of the Japanese version of the Yale-Brown Obsessive-Compulsive Scale. *Psychiatry Clin Neurosci*. 1995;49(2):121-126. doi:10.1111/j.1440-1819.1995.tb01875.x
4. Allen-Crooks R, Challacombe F. Cognitive behavioral therapy for olfactory reference disorder (ORD): A case study. *Journal of Obsessive-Compulsive and Related Disorders*. 2017;13:7–13.
5. Muramatsu K. Patient Health Questionnaire (PHQ-9, PHQ-15) (Japanese version) and. Generalized Anxiety Disorder-7 up to date. Stud. Clin. Psychol. 2014; 7: 35–39.
6. Spitzer RL, Kroenke K, Williams JB. Validation and utility of a self-report version of PRIME-MD: the PHQ primary care study. Primary Care Evaluation of Mental Disorders. Patient Health Questionnaire. *JAMA*. 1999;282(18):1737-1744. doi:10.1001/jama.282.18.1737
7. Spitzer RL, Kroenke K, Williams JB, Löwe B. A brief measure for assessing generalized anxiety disorder: the GAD-7. *Arch Intern Med*. 2006;166(10):1092-1097. doi:10.1001/archinte.166.10.1092
