# Supplementary material for: Cognitive behavioral therapy for a Japanese woman with olfactory reference disorder (ORD) comorbid with schizophrenia: A case study
Source: PCN Rep. 2024 Mar 8;3(1):e179. doi: 10.1002/pcn5.179 (PMC11114287; doi:10.1002/pcn5.179)
Supplement: Supplementary file 2 — Supporting information. [file PCN5-3-e179-s002.docx]

**Table S1. Naomi’s cognitive restructuring sheet.**

| Situation | The new communal living facility that I plan to live in after I leave the hospital comes to mind. |
| --- | --- |
| Emotion/Mood | Depression. Anxiety. |
| Automatic thoughts | 'What if I hate you.'  'What should I do if I can't socialize well.' |
| Evidence | As a child, I was often ridiculed.  I've been told I stink.  Severe anxiety/fear. |
| Rebuttal | ‘In the facility where I have lived so far and the ward where I was hospitalized this time, I have been treated kindly, and I will be able to socialize well without being hated.’ |
| Adaptive thinking | ‘Some people hate me, but not everyone hates me.’ |
| Emotion/Mood change | Slightly relieved depression |
