# Supplementary figures and images for: Cognitive behavioral therapy for a Japanese woman with olfactory reference disorder (ORD) comorbid with schizophrenia: A case study
Source: PCN Rep. 2024 Mar 8;3(1):e179. doi: 10.1002/pcn5.179 (PMC11114287; doi:10.1002/pcn5.179)

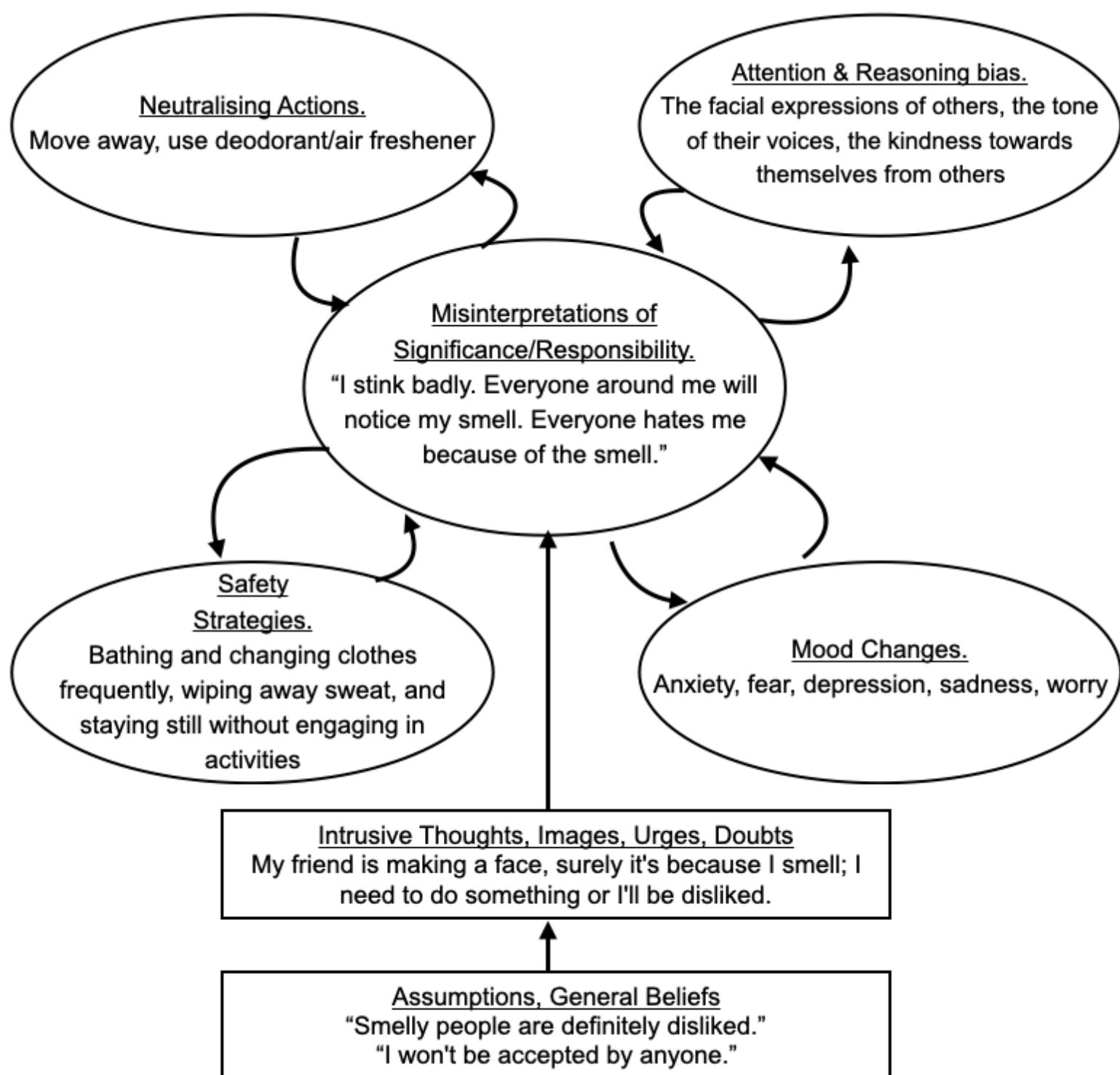

**Fig S1. Cognitive model of Naomi's ORD**

Supplement: Supplementary file 3 — Supporting information. [file PCN5-3-e179-s001.pdf]
